# Case report: Constrictive pericarditis after coronary artery perforation during percutaneous coronary intervention

**DOI:** 10.3389/fcvm.2023.1208376

**Published:** 2023-06-06

**Authors:** Kyung An Kim, Kwan Yong Lee, Byung-Hee Hwang, Do Yeon Kim, Chan Beom Park

**Affiliations:** ^1^ Division of Cardiology, Department of Internal Medicine, Seoul St. Mary’s Hospital, The Catholic University of Korea, Seoul, Republic of Korea; ^2^ Department of Thoracic and Cardiovascular Surgery, Seoul St. Mary’s Hospital, The Catholic University of Korea, Seoul, Republic of Korea; ^3^ Department of Thoracic and Cardiovascular Surgery, Incheon St. Mary’s Hospital, The Catholic University of Korea, Incheon, Republic of Korea

**Keywords:** percutaneous coronary intervention, coronary artery perforation, constrictive pericarditis, echocardiography, pericardiectomy

## Abstract

A 77-year-old man underwent percutaneous coronary intervention (PCI) at the right coronary artery, which was complicated by coronary artery perforation (CAP). After prolonged balloon tamponade proximal to the CAP there was no more contrast extravasation, and the CAP was thought to have resolved. Computed tomography (CT) and echocardiography the following day did not find evidence of continued bleeding, and the patient was discharged. Echocardiograms and chest CT scans obtained one week and two months after PCI detected no remarkable interval change. The patient complained of progressive dyspnea and abdominal distension seven months after PCI however, and echocardiography found an increased amount of pericardial effusion and constrictive physiology. The patient underwent pericardiectomy due to congestive hepatopathy, and progressive dyspnea. The pericardium was thickened and adhesive, and a dark bloody effusion was found. Pathology was unremarkable except for thick fibrosis. After the operation the patient made full recovery, and is stable three years after surgery.

## Introduction

Coronary artery perforation (CAP) is a rare but serious complication after percutaneous coronary intervention (PCI). The incidence is estimated at 0.2% to 0.4% of cases undergoing PCI, with complex PCI procedures such as chronic total occlusions (CTO) posing a higher risk (1). The acute complications of CAP, such as hemopericardium and cardiac tamponade, have been extensively discussed in the literature. However, intermediate- to late- term complications of CAP have seldom been reported. We describe a case of constrictive pericarditis (CP) identified seven months after CAP during initial PCI.

## Case presentation

**Figure 1:**
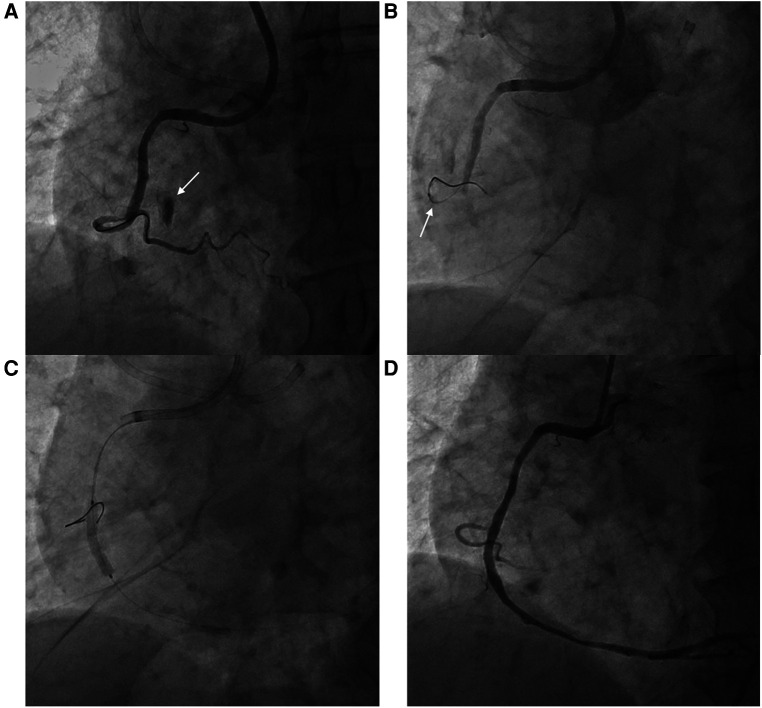
Right ventricular branch perforation during right coronary artery percutaneous coronary intervention. (**A**) Ellis classification type II microchannel perforation during right ventricular branch wiring (arrow) (**B**) Balloon tamponade at the perforated right ventricular branch (arrow) (**C**) Percutaneous coronary intervention at the right coronary artery chronic total occlusion (**D**) Final angiography without residual extravasation at the perforation site.

A 77-year-old man presented to the office complaining of exertional chest pain. His past history was unremarkable. An echocardiogram revealed akinesia at the RCA territory with preserved left ventricular ejection fraction (LVEF) and no pericardial effusion. The patient was admitted for further evaluation, and coronary angiography (CAG) revealed diffuse three-vessel disease, with a right coronary artery (RCA) chronic total occlusion (CTO) with thrombolysis in myocardial infarction (TIMI) grade 0 flow and collateral flow from septal branches. PCI at the RCA CTO lesion was complicated by Ellis classification type II microchannel perforation during right ventricular (RV) branch wiring proximal to the lesion for additional support during CTO wiring (Figure 1). After prolonged balloon inflation at the RCA, no continued contrast extravasation was seen, and the perforation was thought to have resolved. CTO PCI was completed without any further complications, and the patient remained stable throughout the procedure.

Thoracic computed tomography (CT) angiography taken the day after PCI showed no contrast extravasation, and only minimal pericardial effusion was detected. A follow-up echocardiogram was obtained, and there was no visible pericardial effusion and no other remarkable interval changes. The patient was discharged, free from symptoms, and follow-up echocardiograms and chest CT scans obtained one week and two months after PCI detected no remarkable interval change.

Seven months after PCI, the patient complained of progressive dyspnea and abdominal distension and was admitted for further evaluation. The patient's symptoms prompted a follow-up CAG, which revealed no new lesions or in-stent restenosis. Also, no extravasation was noted at the previously perforated RV branch. Echocardiographic findings demonstrated a slightly increased amount of pericardial effusion and inferior vena cava (IVC) plethora, but otherwise no definite constrictive physiology was yet apparent. Laboratory findings were non-specific. Since the overall clinical picture suggested the possibility of progressive CP, he was discharged on 20 mg of prednisolone, 0.6 mg of colchicine, and diuretics.

**Figure 2:**
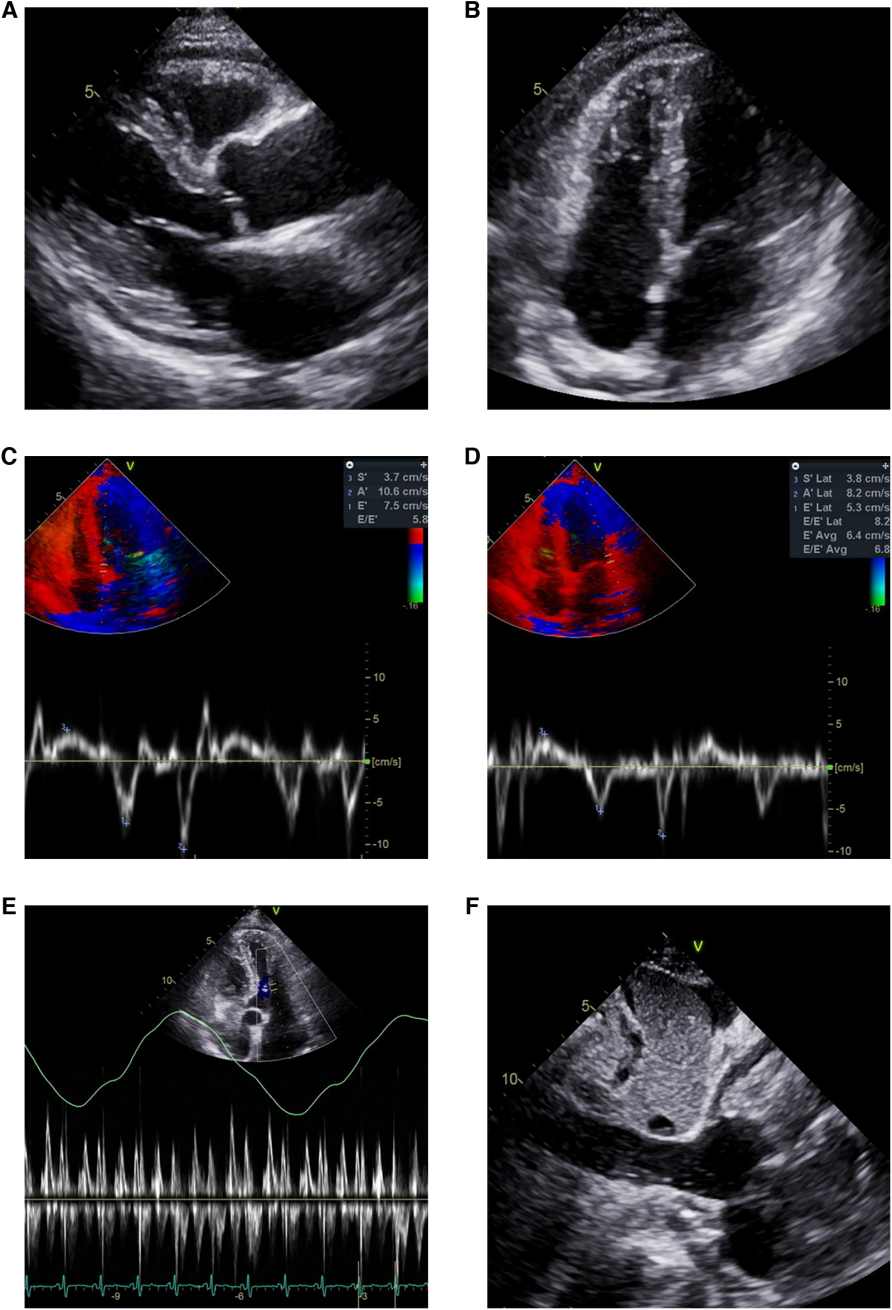
Echocardiogram showing pericardial effusion and constrictive physiology. Small amount of pericardial effusion and thickened pericardium in (**A**) parasternal long axis view and (**B**) modified 4-chamber view. Annulus inversus findings showing (**D**) decreased lateral e’ velocity compared to (**C**) septal e’ velocity. (**E**) Mitral inflow showing significant variations during respiration (**F**) Plethoric inferior vena cava during inspiration.

**Figure 3:**
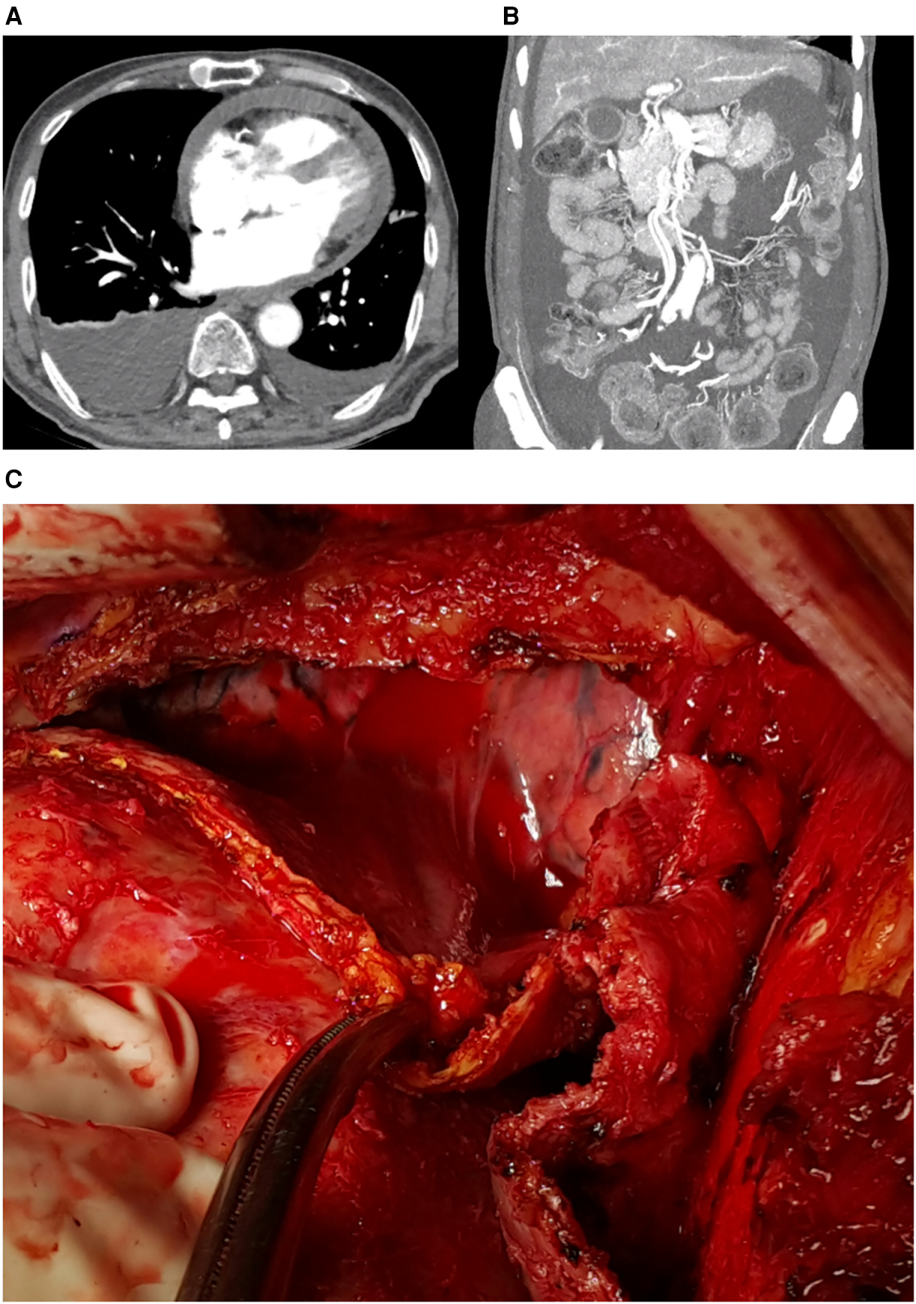
(**A**) Pericardial effusion, pleural effusion, and (**B**) ascites on computed tomograph, (**C**) intraoperative findings: thickened pericardium and hemopericardium.

The patient visited the emergency room two weeks after discharge due to worsening abdominal distension and dyspnea. At presentation, his blood pressure was 95/60 mmHg and heart rate was 70 beats/min. His physical exam was remarkable for grade 2 pitting edema at the lower extremities, a marked jugular venous pulse, pericardial friction rub on ascultation, and a distended abdomen with shifting dullness. Laboratory findings included mildly elevated liver enzymes, with aspartate aminotransferase at 56 U/L and alanine aminotransferase at 64 U/L, and total bilirubin 1.3 mg/dl. A follow-up echocardiogram revealed pericardial thickening and adhesion at the apex and RV anterior wall, along with scanty pericardial effusion. Intraventricular septal bouncing and respiratory mitral and tricuspid valve inflow variability suggested constrictive pericarditis (Figure 2, Supplementary Video S1). Additionally, global hypokinesia resulted in a decreased LVEF of 39%. Large amounts of pleural effusion and ascites were apparent on CT scans of the chest and abdomen, the latter of which was identified as transudate by paracentesis (Figures 3A,B). Unfortunately, due to the patient's instability, right heart catheterization could not be performed.

The patient underwent surgery due to increasing oxygen requirements and hemodynamic instability. Intraoperative findings revealed a thickened (5 mm) and slightly adhesive pericardium without calcifications, and a dark bloody effusion was drained after opening of the pericardium (Figure 3C). Radical pericardiectomy was performed on the anterior, diaphragmatic, and posterior pericardium under cardiopulmonary bypass, and the operation was concluded without major complications. Pathology was negative for tuberculosis, and was unremarkable except for thick fibrosis without evidence of active inflammation. After the operation, the patient experienced significant relief from symptoms such as abdominal distension and dyspnea. Postoperative care was uneventful, and the patient was discharged on diuretics. Minimal remaining septal bouncing was noted on a follow-up echocardiogram two months after surgery, but no other indicators of constrictive physiology were observed. The patient occasionally complains of intermittent chest pain, but is otherwise stable at outpatient follow-up, two years after the surgery. The patient's timeline is presented in Table 1.

**Table 1:** Timeline of treatment.

**Day 1**
CAP develops during PCI and is controlled using prolonged balloon tamponade.
**Day 2**
Minimal pericardial effusion and no continued extravasation at the CAP is found on thoracic CT and echocardiogram.
**Day 7**
Follow-up thoracic CT and echocardiogram demonstrate no interval changes. The patient is stable and is discharged.
**Month 2**
Follow-up thoracic CT and echocardiogram still show minimal pericardial effusion and no extravasation at the CAP site. Patient is free of symptoms.
**Month 7**
The patient complains of progressive dyspnea and abdominal distension. Echocardiography demonstrates a slightly increased amount of pericardial effusion, but no extravasation is noted on follow-up CAG. The patient is discharged on steroids and colchicine.
**Month 8**
The patient's symptoms worsen. Follow-up echocardiogram reveals findings of constrictive pericarditis. Pericardiectomy is performed, after which the patient improves. He is discharged uneventfully.
**Month 10**
No remaining constrictive physiology is noted on follow-up echocardiogram.
**Year 2**
The patient is stable at outpatient follow-up.

## Discussion

The most common identifiable causes of CP in the modern era are prior cardiac surgery, infections such as tuberculosis, and post-radiation (2). Hemopericardium is an infrequent cause of CP and usually appears as a mid- to late-term complication, while it has also been implicated in the development of CP after cardiac surgery (3). Fibrosis developing from pericardial irritation from pooled blood and serosal injury has been suggested as a possible cause, although the precise causes remain unknown (3–5).

Bonello et al. (6) previously reported a case of CP occurring six months after a type III CAP and resulting in hemopericardium, sealed using a graft stent and after which there was no continued hemopericardium. Mohamed et al. reported a case of CP diagnosed five months after PCI with no obvious coronary perforation during the procedure, where no pericardial effusion was identified during the course of follow-up (7). Based on the characteristics of the reported cases, we hypothesize that post-CAP CP shares features with post-cardiac surgery CP. In contrast to CP from other causes, in post-cardiac surgery CP there is minimal adhesion or calcification, and the constriction is instead usually due to thickening and fibrosis of the pericardium, (3) findings which were observed in our case and also previous reports of post-CAP CP. There is evidence for the role of antimyocardial antibody titers and pericardial damage in post-cardiac injury syndrome, and it may be that pro-inflammatory conditions and autoimmune factors were implicated in this case also (8). The time to development in post-surgery CP is unpredictable and ranges from one month to several years after surgery (3). Varying intensities of inflammatory and fibrotic responses may be responsible for this variation. Although our patient showed signs of CP seven months after PCI, which is similar to the previously mentioned post-CAP cases, it can be expected that other cases of post-CAP CP may present with a completely different timeline.

On the other hand, the possibility of delayed coronary artery extravasation after the initial perforation during PCI cannot be ruled out, although serial follow up CT scans and echocardiograms did not show an increase in pericardial effusion up to two months after the initial event. If delayed extravasation was present, it may have contributed to the development of CP, as well as causing hemopericardium. Alternatively, an effusive-constrictive pericarditis may have developed by independent, unknown mechanisms after the initial perforation, but this seems unlikely given the low prevalence of effusive-constrictive pericarditis.

There are a number of management approaches for treating CAP (1). For less severe cases, simple observation with frequent echocardiographic monitoring for hemopericardium assessment during PCI may suffice. Prolonged balloon dilatation to block the blood flow to the perforation site, as used in our case, can be applied easily and is widely used. For more severe cases, polytetrafluoroethylene-covered graft stents can be used for definitive treatment. However, in clinical practice, it can be challenging to determine if extravasation has ceased or if additional measures are needed. Our case illustrates that CAP can lead to subsequent unexpected complications, and underlines the need for both adequate bleeding control and careful monitoring.

Because pericardiectomy has the potential to provide relief of symptoms and improve prognosis, early recognition of CP is necessary. Patients with CAP in particular should be monitored using appropriate diagnostic testing, as this case illustrates. The typical patient will display signs and symptoms of right heart failure, with peripheral edema, abdominal distension, and elevated jugular venous pressure. Signs of hepatic dysfunction, such as elevated bilirubin and hypoalbuminemia, have been associated with increased mortality in CP patients undergoing pericardiectomy (2). Multimodality imaging techniques, with echocardiography as a first choice, are necessary for diagnosis. Characteristic echocardiographic findings of CP include diastolic septal bounce, plethoric IVC, and respiratory mitral and tricuspid inflow variation (9).

## Conclusion

We report a case of CP which developed seven months after PCI for a CTO lesion complicated by CAP. The reason for the development of CP after CAP is unclear, but may be due to pericardial inflammation and fibrosis from contact with blood, which shares similarities with the etiology of post-surgery CP. Patients with CAP during PCI should be regularly monitored, and CP should be suspected in patients who present with peripheral edema, abdominal distension, and laboratory findings suggesting liver cirrhosis. Definite diagnosis can be made by imaging modalities including echocardiography, and pericardiectomy has the potential to relieve symptoms and improve prognosis.

## Data Availability

The original contributions presented in the study are included in the article/Supplementary Material, further inquiries can be directed to the corresponding author.

## Supplementary material

Supplementary Video Echocardiogram demonstrating findings of constrictive pericarditis.

## References

[1] Shimony A Joseph L Mottillo S Eisenberg MJ. Coronary artery perforation during percutaneous coronary intervention: a systematic review and meta-analysis. Can J Cardiol. (2011) 27:843–50. 10.1016/j.cjca.2011.04.014 21862280

[2] George TJ Arnaoutakis GJ Beaty CA Kilic A Baumgartner WA Conte JV. Contemporary etiologies, risk factors, and outcomes after pericardiectomy. Ann Thorac Surg. (2012) 94:445–51. 10.1016/j.athoracsur.2012.03.079 22621875 PMC3610598

[3] Gaudino M Anselmi A Pavone N Massetti M. Constrictive pericarditis after cardiac surgery. Ann Thorac Surg. (2013) 95:731–6. 10.1016/j.athoracsur.2012.08.059 23266135

[4] Cameron J Oesterle SN Baldwin JC Hancock EW. The etiologic spectrum of constrictive pericarditis. Am Heart J. (1987) 113:354–60. 10.1016/0002-8703(87)90278-X 3812191

[5] Cliff WJ Grobety J Ryan GB. Postoperative pericardial adhesions. The role of mild serosal injury and spilled blood. J Thorac Cardiovasc Surg. (1973) 65:744–50. 10.1016/S0022-5223(19)40710-1 4696874

[6] Bonello L Paule P Quilici J Lambert M Fourcade L Bonnet JL. An unusual mid term complication of coronary rupture. Int J Cardiol. (2005) 104:119–21. 10.1016/j.ijcard.2005.06.014 16014314

[7] Mohamed HA Korkola S. A modern cause of an old disease. Constrictive pericarditis after percutaneous coronary intervention: a case report. Int J Angiol. (2008) 17:106–8. 10.1055/s-0031-1278291 22477398 PMC2728418

[8] Imazio M Hoit BD. Post-cardiac injury syndromes. An emerging cause of pericardial diseases. Int J Cardiol. (2013) 168:648–52. 10.1016/j.ijcard.2012.09.052 23040075

[9] Adler Y Charron P Imazio M Badano L Baron-Esquivias G Bogaert J ESC guidelines for the diagnosis and management of pericardial diseases: the task force for the diagnosis and management of pericardial diseases of the European society of cardiology (ESC)endorsed by: the European association for cardio-thoracic surgery (EACTS). Eur Heart J. (2015) 2015(36):2921–64. 10.1093/eurheartj/ehv318 PMC7539677 26320112

